# Wireless Powered Encoding and Broadcasting of Frequency Modulated Detection Signals

**DOI:** 10.1109/access.2020.3035938

**Published:** 2020-11-04

**Authors:** WEI QIAN, XIN YU, CHUNQI QIAN

**Affiliations:** 1 Department of Electrical and Computer Engineering, Michigan State University, East Lansing, MI 48824, USA; 2 Martinos Center for Biomedical Imaging, Massachusetts General Hospital, Charlestown, MA 02129, USA; 3 Department of Radiology, Michigan State University, East Lansing, MI 48824, USA

**Keywords:** Frequency modulation, nonlinear circuits, oscillator, inductive power transmission, magnetic resonance

## Abstract

Wireless transmission of locally detected RF signals is necessary for long-term operation of batteryless and embedded transducers. To improve signal transmission efficiency over larger distances, multi-stage circuits were employed to down-convert RF signals before encoding them onto the emitted carrier wave. Such multi-stage arrangement had complicated design and high-power consumption. Here, a compact and low-power wireless modulator is introduced to directly encode input RF signals onto its oscillation carrier wave. The modulator consists of a double frequency parametric resonator that is overlaid with a single frequency passive resonator to create three resonance modes. By properly adjusting the substrate thickness between resonators, the highest resonance frequency is tuned to approximately the sum of lower two resonance frequencies, enabling efficient conversion of wireless pumping power into sustained oscillation currents. When an input RF signal is present with a certain frequency offset, the oscillation signal can be frequency modulated by the input signal to create multiple modulation sidebands separated by the offset frequency. The frequency encoded carrier wave can transmit MRI signals over larger distance separations to maintain constant image sensitivity, making the modulator useful to improve the remote detectability of miniaturized implantable and interventional devices.

## I. INTRODUCTION

Wireless signal transducers are particularly advantageous to improve the long-term applicability of physiological sensors [1]–[3] embedded inside confined body cavities. When an external receiver coil is close enough to the embedded transducer, passive inductive coupling is normally utilized to wirelessly relay encoded signals. For communications beyond the near field region, these embedded transducers can be wirelessly powered to convert physiological parameters into voltage signals that can modulate the amplitude [4] or frequency [5] of the emitted carrier wave using analog or digital schemes. However, these wireless transducers are mainly effective for input frequencies that are much lower than the carrier frequency. To encode input signals in the radio frequency range, RF signals are usually down converted to below the MHz range [6] before being digitized to modulate a GHz carrier. Alternatively, RF input signals can be directly digitized and encoded onto carriers waves with increasingly higher frequencies, from the millimeter [7] to the optical [8] range. These multi-stage schemes require complicated circuits with larger size and higher power consumption. Even though high performance laser diodes [9] or electro-optical modulators [10]–[14] can obviate the need for ADC conversion thus reducing power consumption, inefficient transmission of light through biological tissues mandate the use of optical fibers that are still not suitable for long-term insertion into enclosed cavities. Therefore, it will be ideal to combine down-conversion and signal encoding into a single stage, for simpler circuit design and lower power consumption. Such a compact and integrated transducer can be useful to encode locally detected ultrasound signals onto EM waves that can penetrate the skull [15], or to increase the effective coupling distance of implantable MRI detectors [16]–[18] beyond the near field region. Removal of hard-wired connections will also be useful to improve the operation flexibility of interventional detectors [19]–[21].

In this work, we are going to construct a wirelessly powered frequency modulator that can combine down conversion and signal encoding into a single stage. The core component of this compact modulator is a double-frequency parametric resonator whose second resonance mode is created by bridging the virtual voltage grounds of its first resonance mode. The double-frequency parametric resonator is coaxially overlaid with another single frequency resonator to create a third resonance mode. By properly adjusting the substrate thickness between these two resonators, the third resonance frequency is tuned to approximately the sum of two lower resonance frequencies. When activated by a pumping field near the highest resonance frequency, the coupled resonator pair can utilize its nonlinear capacitance to convert wireless pumping power into oscillation currents circulating near its two lower resonance frequencies. In the presence of an input RF signal that is deviated from the oscillation signal by an offset frequency, the oscillation signal is frequency modulated by the input signal to create multiple sidebands that are separated by the offset frequency. As a result, this frequency modulated oscillation signal can be utilized to transmit locally detected MRI signals with improved sensitivity over large distance separations, until the oscillation signal is detected by an external receiver coil that is interfaced with a conventional MRI scanner. After frequency demodulation of the received oscillation signal, MR images can be reconstructed from 2D Fourier transform to maintain constant signal-to-noise ratio even though the parametric resonator is displaced from the external detector by 11-fold of its own dimension (corresponding to a 34-dB transmission attenuation with respect to a cable-connected surface coil). The wireless modulator requires only ~ 9 dBm of pumping power on the activation antenna that is separated by 4-fold the modulator’s largest dimension, making itself particularly suitable to improve the detection sensitivity of implantable or interventional MRI detectors, whose small dimension and large penetration depth will prevent efficient signal transmission by passive coupling alone. Complementary to other types of RF transducers (Table 1), our wirelessly powered frequency modulator may also operate in conjunction with embedded perpetual sensors [22] to encode multiple types of environmental signals [23] in the radio frequency range.

## II. OPERATION PRINCIPLE

**FIGURE 1.**
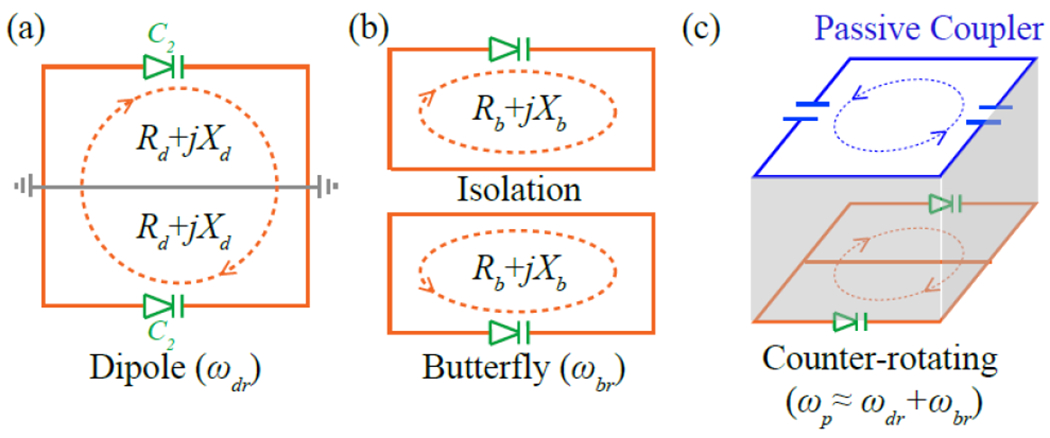
The double frequency parametric resonator can be implemented as a rectangular resonator with identical nonlinear capacitors (*C*_2_) symmetrically distributed on its top and bottom edges. (a) When the circuit’s left and right edges are connected by a continuous conductor in their centers, the dipole mode resonance can be analyzed as two identical half circuits that are grounded in the center. Each half circuit has an effective impedance of *R_d_*+*jX_d_* with current flows in the same directions. (b) The circuit’s butterfly mode resonance mode can be considered as two half circuits that are electrically isolated in the center. Both half circuits have current flows in opposite directions; each half circuit has effective impedance of *R_b_*+*jX_b_*. (c) When the double frequency parametric resonator is coaxially overlaid with another single frequency passive resonator, a third resonance mode is created to allow opposite current flows in both resonators. By properly adjusting the substrate thickness (shown in grey) between the coupled resonators, the highest resonance frequency becomes approximately the sum of the two lower resonance frequencies, enabling effective conversion of pumping currents induced in the counter-rotating mode into sustained oscillation currents in the dipole and butterfly modes.

A parametric oscillator is a nonlinear multi-frequency resonator that can convert wireless pumping power into sustained oscillation currents. As shown in Fig. 1a, the resonator can be implemented as a rectangular inductor that is serially connected to two varactors on its top and bottom edges, creating a dipole mode at resonance frequency *ω_dr_*. When the virtual voltage grounds of the dipole mode are connected by a continuous conductor in the center, the resonator will have a butterfly mode at resonance frequency *ω_br_* (Fig. 1b), where current flows in both circuit halves are opposite. When the circuit is inductively activated by an external pumping field at approximately the sum frequency *ω_p_* = *ω_dr_* + *ω_br_*, the pumping power is effectively converted into sustained oscillation currents in both the dipole and butterfly modes. This power conversion process is facilitated by the voltage-dependent capacitance of both varactors:

(1) $$C_{2}\approx C_{20}\left(1+\lambda_{2}V_{p}/\phi_{2}\right)$$

where *C*_20_ is the junction capacitance at zero bias, λ_2_ is a device constant describing the abruptness of charge distribution across the depletion layer, *ϕ*_2_ is the junction potential of the depletion layer, *V_p_* is the effective pumping voltage across each diode.

Although parametric oscillators were implemented for pressure [24] and voltage [5] sensing, those circuits were only utilized for quasi-static signal sensing. In this work, we will extend the applicability of coupled parametric resonator towards RF signal sensing. Suppose an input voltage source *ζ_d_* has a frequency that is slightly deviated from the dipole mode oscillation frequency *ω_d_* by Δ*ω_d_*, the voltage-current relation [24] at *ω_d_* can be modified to the following approximation:

(2) $$\xi_{d}e^{j\Delta \omega_{d}t}\approx I_{d}\left(R_{d}+jX_{d}\right)+\frac{\lambda_{2}\phi_{2}V_{p}I_{b}^{*}}{jC_{20}\omega_{b}\left(\phi_{2}^{2}-\lambda_{2}^{2}{\left|V_{p}\right|}^{2}\right)}$$

where *R_d_*+*jX_d_* is the effective impedance of each half circuit at *ω_d_* (as shown in Fig. 1a). Similarly, the electromotive force *ζ_b_* at *ω_b_* (the butterfly mode oscillation frequency) satisfies the following approximation:

(3) $$\xi_{b}^{*}e^{-j\Delta \omega_{b}t}\approx \frac{j\lambda_{2}\phi_{2}V_{p}^{*}I_{d}}{C_{20}\omega_{d}\left(\phi_{2}^{2}-\lambda_{2}^{2}{\left|V_{p}\right|}^{2}\right)}+I_{b}^{*}{\left(R_{b}+jX_{b}\right)}^{*}$$

By combining Eqns. (2) and (3), we obtain

(4) $$I_{b}^{*}=\frac{\left(\xi_{d}e^{j\Delta \omega_{d}t}-I_{d}\left(R_{d}+jX_{d}\right)\right)jC_{20}\omega_{b}\left(\phi_{2}^{2}-\lambda_{2}^{2}{\left|V_{p}\right|}^{2}\right)}{\lambda_{2}\phi_{2}V_{p}}\;=\frac{\xi_{b}^{*}e^{-j\Delta \omega_{b}t}+\frac{I_{d}\lambda_{2}\phi_{2}V_{p}^{*}}{jC_{20}\omega_{d}\left(\phi_{2}^{2}-\lambda_{2}^{2}{\left|V_{p}\right|}^{2}\right)}}{\left(R_{b}-jX_{b}\right)}$$

When there is no input *e.m.f.* for the butterfly mode, i.e. $\xi_{b}^{*}=0$, Eq. (4) is equivalated to

(5) $$\xi_{d}e^{j\Delta \omega_{d}t}\left(R_{b}-jX_{b}\right)=I_{d}\left(R_{d}+jX_{d}\right)\left(R_{b}-jX_{b}\right)-\frac{I_{d}\lambda_{2}^{2}\phi_{2}^{2}{\left|V_{p}\right|}^{2}}{C_{20}^{2}\omega_{d}\omega_{b}{\left(\phi_{2}^{2}-\lambda_{2}^{2}{\left|V_{p}\right|}^{2}\right)}^{2}}$$

Equating the real parts on both sides of Eq. (5):

(6) $$\xi_{d}\left(R_{b}\;\cos\left(\Delta \omega_{d}t\right)+X_{b}\;\sin\left(\Delta \omega_{d}t\right)\right)\;=I_{d}\left(R_{d}R_{b}+X_{d}X_{b}\right)-\frac{I_{d}\lambda_{2}^{2}\phi_{2}^{2}{\left|V_{p}\right|}^{2}}{C_{20}^{2}\omega_{d}\omega_{b}{\left(\phi_{2}^{2}-\lambda_{2}^{2}{\left|V_{p}\right|}^{2}\right)}^{2}}\approx I_{d}\cdot 0$$

The second line in Eq. (6) is mandated to approximate zero because circuit oscillation requires the circulating current *I_d_* to be non-negligible even though the input signal *ξ_d_* is close to zero. This relation can be satisfied by gradually increasing the externally applied pumping power until the effective pumping voltage *V_p_* across both varactors is above a certain threshold. Besides the pumping power requirement, the modulated oscillation frequency can be obtained by equating the imaginary parts on both sides of Eq. (5):

(7) $$\xi_{d}\left(jR_{b}\sin\left(\Delta \omega_{d}t\right)-jX_{b}\cos\left(\Delta \omega_{d}t\right)\right)=+I_{d}\left(jX_{d}R_{b}-jR_{d}X_{b}\right)$$

Or equivalently

(8) ξd(Rbsin(Δωdt)Xb−cos(Δωdt))=ξdRbsin(Δωdt)2(ωb−ωbr)Lb−ξdcos(Δωdt)=IdRd(XdRbXbRd−1)≈IdRd((2(ωd−ωdr)LdRd)/(2(ωb−ωbr)LbRb)−1)

Eq. (8) describes the relation between the RF input voltage *ξ_d_* and the circuit oscillation frequencies *ω_d_* and *ω_b_* that are slightly deviated from *ω_dr_* and *ω_br_* (i.e. the dipole and butterfly mode resonance frequencies respectively). By plugging *ω_b_* = *ω_p_* − *ω_d_* into Eq. (8) and solving for *ω_d_*:

(9) $$\omega_{d}=\frac{2I_{d}L_{b}R_{d}\omega_{br}-2I_{d}L_{d}R_{b}\omega_{dr}-2I_{d}L_{b}R_{d}\omega_{p}-2L_{b}\left(\omega_{br}-\omega_{p}\right)\xi_{d}\;\cos\left(\Delta \omega_{d}t\right)-R_{b}\xi_{d}\;\sin\left(\Delta \omega_{d}t\right)}{-2L_{d}I_{d}R_{b}-2L_{b}I_{d}R_{d}+2L_{b}\xi_{d}\;\cos\left(\Delta \omega_{d}t\right)}\;\approx \frac{L_{b}R_{d}\omega_{br}-L_{d}R_{b}\omega_{dr}-L_{b}R_{d}\omega_{p}}{-L_{d}R_{b}-L_{b}R_{d}}+\frac{\left(\omega_{br}-\omega_{p}\right)\xi_{d}\;\cos\left(\Delta \omega_{d}t\right)}{L_{d}I_{d}R_{b}/L_{b}+I_{d}R_{d}}\;\equiv \omega_{d0}+2\pi k_{f}\xi_{d}\;\cos\left(\Delta \omega_{d}t\right)$$

Approximation in the second line of Eq. (9), as shown at the bottom of the page, is obtained under the common condition that the resonator has high quality factor, i.e. *L_b_*(*ω_br_* − *ω_p_*) ≫ *R_b_*, and that the oscillation induced voltage is much larger than the input electromotive force, i.e. *I_d_R_d_* ≫ *ξ_d_*. According to the third line of Eq. (9), the oscillation frequency *ω_d_* is linearly modulated by the input signal *ξ_d_* around a center frequency *ω*_*d*0_, and the magnitude of frequency deviation can be expressed as:

(10) $$2\pi k_{f}\xi_{d}=\frac{\left(\omega_{br}-\omega_{p}\right)\xi_{d}}{\left(L_{d}I_{d}R_{b}/L_{b}+I_{d}R_{d}\right)}$$

where *k_f_* is the modulator’s frequency sensitivity. Eq. (10) indicates that the frequency deviation is independent of the frequency offset Δ*ω_d_* of the input signal. By integrating the instantaneous frequency in Eq. (9) to obtain the phase, the oscillation signal at time point *t* can be represented as:

(11) $$I_{d}\;\exp\left(j\omega_{d0}t+j2\pi k_{f}\xi_{d}\int_{0}^{t}\cos\left(\Delta \omega_{d}t\right)dt\right)\;=I_{d}\;\exp\left(j\omega_{d0}t+\frac{j2\pi k_{f}\xi_{d}}{\Delta \omega_{d}}\sin\left(\Delta \omega_{d}t\right)\right)\;\equiv I_{d}\;\exp\left(j\omega_{d0}t+j\beta \;\sin\left(\Delta \omega_{d}t\right)\right)\;=I_{d}\sum_{n=-\infty }^{n=\infty }J_{n}\left(\beta \right)\;\exp\left(j\omega_{d0}t+j2\pi n\Delta \omega_{d}t\right)$$

where *β* is the frequency deviation ratio and *J_n_*(*β*) is Bessel function of the first kind. Therefore, the spectrum of frequency modulated oscillation signal is the superposition of multiple peaks at *ω*_*p*0_+*n*Δ*ω_d_*. Moreover, the relative intensity of the first sideband with respect to the center band is:

(12) $$\frac{J_{1}\left(\beta \right)}{J_{0}\left(\beta \right)}\approx \frac{\beta }{2}\equiv \frac{2\pi k_{f}\xi_{d}}{2\Delta \omega_{d}}=\frac{\left(\omega_{br}-\omega_{p}\right)\xi_{d}}{2\left(L_{d}I_{d}R_{b}/L_{b}+I_{d}R_{d}\right)\Delta \omega_{d}}$$

Besides analysis of its spectral feature in the frequency domain, the oscillation signal expressed in Eq. (11) can also be demodulated in the time domain by derivatizing its phase angle, thus retrieving the magnitude of frequency deviation for a specific input signal, as specified in Eq. (10).

**FIGURE 2.**
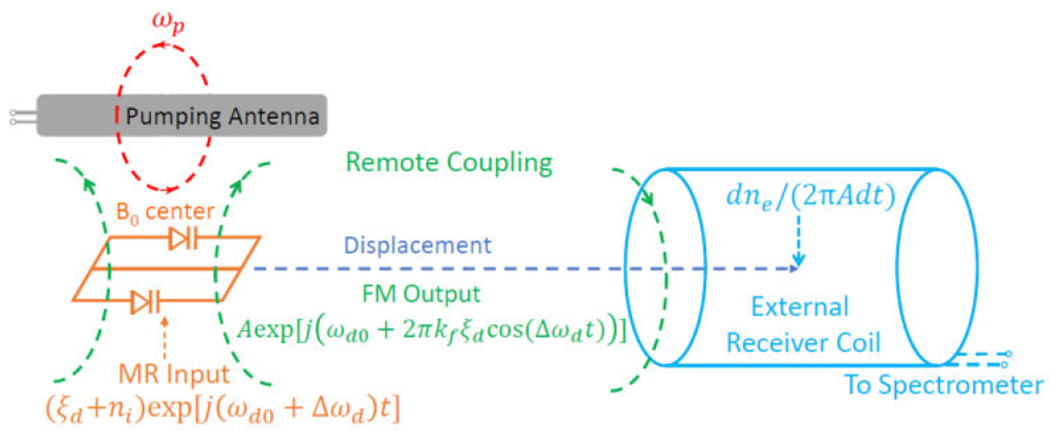
When activated by the pumping field (red), the parametric oscillator (orange) will encode locally detected MRI signals *ξ_d_* and internal noise *n_i_* onto its oscillation signal that is detected by the external receiver coil (cyan) via remote inductive coupling, where the external noise *n_e_* also affects the retrieved spectra baseline.

In order to estimate the noise contribution to the demodulated output, it is necessary to consider both the internal noise (that is detected by the modulator) and the external noise (that resides on the external receiver coil). In the time domain, the time-dependent internal noise voltage *n_i_*(*t*) contributes to instantaneous frequency shift of oscillation signal according to Δ*f_i_*(*t*) = *k_f_n_i_*(*t*). Because *k_f_* is invariant to input signal offset as indicated by Eq. (10), the Fourier transform of Δ*f_i_*(*t*) is

(13) $$\hat{f}\left(k_{f}n_{i}\left(t\right)\right)=k_{f}N_{i}$$

where *N*_*i*_ is the frequency domain spectrum of the modulator’s noise voltage that is normally frequency independent. Eq. (13) describes the “internal” noise from the modulator that will be frequency encoded onto the oscillation signal. Subsequently, this oscillation signal will be detected by the external receiver coil that is remotely coupled to the modulator (Fig. 2).

For very weak coupling condition, there is also non-negligible noise contribution from the external receiver coil, whose instantaneous noise voltage *n_e_*(*t*) will introduce an additional phase fluctuation according to *ϕ_e_*(*t*) = *n_e_*(*t*)/*A* where *A* is the oscillation voltage induced on the external receiver coil. As a result, the equivalent noise in the frequency domain can be obtained by Fourier transform of the time-domain derivative of phase fluctuation:

(14) $$\hat{f}\left(\frac{d\phi_{e}\left(t\right)}{2\pi \;dt}\right)=\hat{f}\left(\frac{dn_{e}\left(t\right)}{2\pi \;Adt}\right)=\frac{\Delta f_{d}N_{e}}{A}$$

where Δ*f_d_* is the frequency offset of the input signal from the oscillation signal and *N_e_* is the frequency domain spectrum of the noise voltage originating from the external receiver coil. *N_e_* is normally frequency independent, but its contribution to the FM demodulated output is dependent on the frequency offset Δ*f_d_*, as indicated by Eq. (14). By combining the contribution from Eqns. (13) and (14), the signal-to-noise power ratio is

(15) $$\frac{P_{S}}{P_{N}}=\frac{k_{f}^{2}{\left|\xi_{d}\right|}^{2}}{k_{f}^{2}N_{i}^{2}+{\left(\Delta f_{d}N_{e}/A\right)}^{2}}$$

(16) $$L=\frac{\mu_{0}}{\pi }\left[\begin{array}{c} \left(A+B\right)\;\ln\;\left(2AB/W\right)-A\;\ln\;\left(A+\sqrt{A^{2}+B^{2}}\right)-B\;\ln\;\left(B+\sqrt{A^{2}+B^{2}}\right)\\ -\left(A+B\right)/2+2\left(\sqrt{A^{2}+B^{2}}\right)+0.447W \end{array}\right]$$

Eq. (15) indicates that when the oscillation voltage *A* induced on the external receiver coil is sufficiently large, Δ*f_d_N_e_*/*A* will be much smaller than *k_f_N_i_*, making the noise baseline almost flat in the demodulated spectrum. But when *A* is small due to very large transmission attenuation, the frequency dependent noise floor can no longer be neglected.

## III. MATERIALS AND METHODS

### A. ELECTROMAGNETIC SIMULATION

**FIGURE 3.**
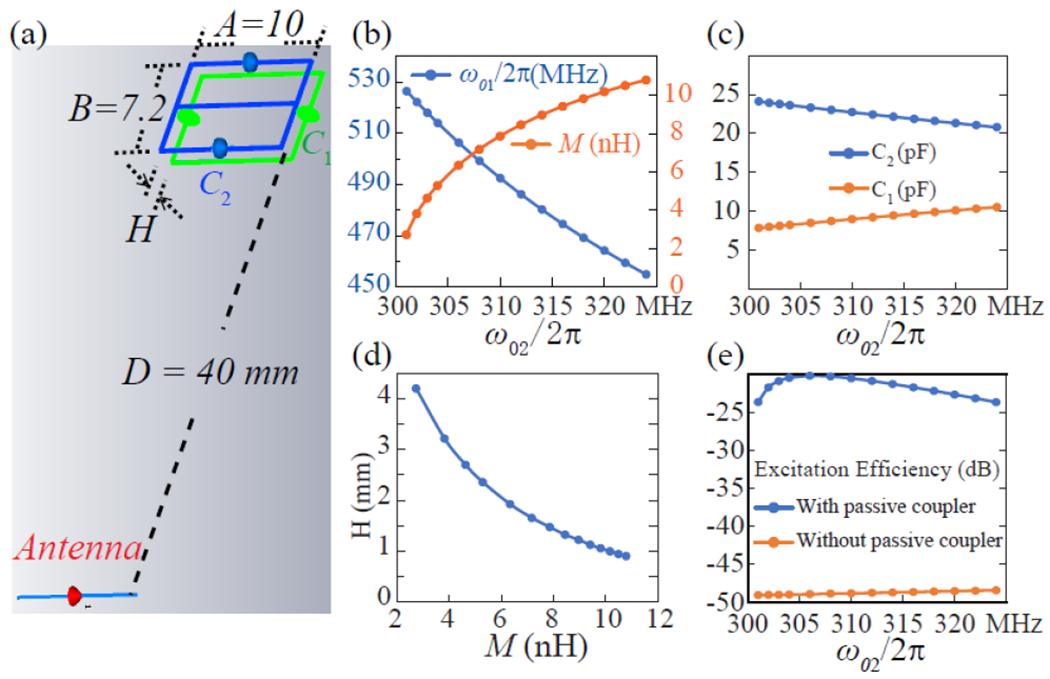
(a) The 3D model of a wireless modulator created by overlaying a parametric resonator (blue) with a passive coupler (green). Both resonators consist of a rectangular loop inductor split by two capacitors. The modulator is activated by an antenna (red) separated by 40 mm, which is 4-fold the modulator’s largest dimension. (b) By specifying its circuit dimension, the parametric resonator is tuned by two serial connected varactors to a frequency *ω*_02_ that is slightly above the MR frequency. Subsequently, the resonance frequency *ω*_01_ of the passive coupler and the mutual inductance *M* between these two resonators are also determined. (c) To tune the parametric resonator to the desired frequency of *ω*_02_, two serial capacitors of *C*_2_ values are required. Similarly, to tune the passive resonator to the desired frequency of *ω*_01_, two serial capacitors of *C*_1_ values are required. (d) Each mutual inductance of *M* requires the two resonators to be separated by a gap size of *H* that can be directly incorporated into the 3D model in (a). (e) The blue curve shows the excitation efficiency of the coupled resonator pair that is separated from the antenna by 4-fold the modulator’s own dimension. The orange curve shows the excitation efficiency of a parametric in its stand-alone configuration.

To design a wireless modulator, the first step is to specify the dimension of a double frequency parametric resonator (blue lines in Fig. 3a) whose self-inductance is [25], (16) as shown at the bottom of the page, where *A* and *B* are the rectangle’s side lengths, *W* is the width of the conductor strip. For a resonator with *A* = 10 mm and *B* = 7.2 mm, its effective inductance is *L* = 23.2 nH. When the rectangle’s conductor gaps are filled by properly chosen varactors, its dipole mode resonance *ω*_02_ should be tuned to a frequency that is slightly above the MR frequency. Meanwhile, existence of the continuous center conductor will introduce another butterfly mode resonating at ~77% the resonance frequency of the dipole mode.

To improve the circuit’s coupling efficiency with the activation antenna, a passive coupler is coaxially overlaid with the parametric resonator to locally concentrate the pumping field. This passive coupler has identical dimension as the parametric resonator but is tuned to a higher frequency at *ω*_01_. Overlapping these two resonators will not affect the butterfly mode frequency of the parametric resonator, but will create shifted dipole mode frequencies that can be estimated from the following equation [24]:

(17) $$0\approx j\omega L-\frac{j\omega_{01}^{2}L}{\omega }+\frac{\omega^{2}M^{2}}{j\omega L-j\omega_{02}^{2}L/\omega }$$

where *ω*_01_ and *ω*_02_ are the resonance frequencies of the passive coupler and the parametric resonator in their stand-alone configuration. *M* is the mutual inductance of overlapped inductors that can be estimated from [25]:

(18) $$M=2\left(M_{11^{\prime }}+M_{22^{\prime }}-M_{13^{\prime }}-M_{24^{\prime }}\right)\;=2\left\{\begin{array}{c} -\frac{\mu_{0}A}{2\pi }\left[\ln\left(\frac{A}{\sqrt{H^{2}+B^{2}}}+\sqrt{1+\frac{A^{2}}{H^{2}+B^{2}}}\right)+\frac{\sqrt{H^{2}+B^{2}}}{A}-\sqrt{\frac{H^{2}+B^{2}}{A^{2}}+1}\right]\\ -\frac{\mu_{0}B}{2\pi }\left[\ln\left(\frac{B}{\sqrt{H^{2}+A^{2}}}+\sqrt{1+\frac{B^{2}}{H^{2}+A^{2}}}\right)+\frac{\sqrt{H^{2}+A^{2}}}{B}-\sqrt{\frac{H^{2}+A^{2}}{B^{2}}+1}\right]\\ +\frac{\mu_{0}A}{2\pi }\left[\ln\left(\frac{A}{H}+\sqrt{1+\frac{A^{2}}{H^{2}}}\right)+\frac{H}{A}-\sqrt{\frac{H^{2}}{A^{2}}+1}\right]\\ +\frac{\mu_{0}B}{2\pi }\left[\ln\left(\frac{B}{H}+\sqrt{1+\frac{B^{2}}{H^{2}}}\right)+\frac{H}{B}-\sqrt{\frac{H^{2}}{B^{2}}+1}\right] \end{array}\right\}$$

where *H* is the substrate thickness between the coupled resonator pair. To evaluate the relationship between dipole mode frequencies, Eq. (17) can be rearranged as:

(19) $$\frac{L^{2}}{\omega^{4}}-\frac{L^{2}}{\omega^{2}}\left(\frac{1}{\omega_{01}^{2}}+\frac{1}{\omega_{02}^{2}}\right)+\frac{L^{2}-M^{2}}{\omega_{01}^{2}\omega_{02}^{2}}=0$$

where *ω*_01_ and *ω*_02_ are resonance frequencies of uncoupled resonators. According to Vedic theorem, Eq. (19) should have two solutions for 1/*ω*_2_ that correspond to the inverse square of the lower coupled frequency $1/\omega_{L}^{2}$ and the inverse square of the higher coupled frequency $1/\omega_{H}^{2}$:

(20) $$\frac{1}{\omega_{L}^{2}}+\frac{1}{\omega_{H}^{2}}=\frac{1}{\omega_{01}^{2}}+\frac{1}{\omega_{02}^{2}}$$

(21) $$\frac{1}{\omega_{L}^{2}\omega_{H}^{2}}=\frac{1-M^{2}/L^{2}}{\omega_{01}^{2}\omega_{02}^{2}}$$

Proper adjustment of the mutual inductance *M* between coupled resonators should allow the higher coupled frequency *ω_H_* to be the sum of the lower coupled frequency *ω_L_* and the butterfly mode frequency *ω_br_* of the parametric resonator. Meanwhile, the lower coupled frequency *ω_L_* should be equal to the MR frequency:

(22) $$\omega_{H}-\omega_{L}=\omega_{br}\approx 0.77\omega_{01}$$

(23) $$\omega_{L}=\omega_{MR}$$

By plugging Eqns. (22) and (23) into Eq. (20), the required resonance frequency *ω*_01_ of the passive coupler can be estimated as:

(24) $$\omega_{01}=1/\sqrt{1/{\left(\omega_{MR}+0.77\omega_{02}\right)}^{2}+1/\omega_{MR}^{2}-1/\omega_{02}^{2}}$$

Therefore, once the resonance frequency *ω*_02_ of the parametric resonator is known, the required resonance frequency *ω*_01_ of the passive coupler can be estimated from Eq. (24), and the required mutual inductance *M* between these two resonators can be estimated from Eq. (21). To incorporate these parameters into the circuit model in Fig. 3a, the required serial capacitance for both resonators can be calculated as $C_{2}=2/\left(\omega_{02}^{2}L\right)$ and $C_{1}=2/\left(\omega_{01}^{2}L\right)$, as shown in Fig. 3c. Meanwhile, the required substrate thickness *H* between these two resonators can be estimated from Eq. (18) as shown at the bottom of the next page, to match the corresponding mutual inductance *M* in Fig. 3d. When unit current is applied on the dipole antenna that is separated from the modulator by 4-fold of the modulator’s own dimension, the current *I_p_* induced inside the parametric resonator can be numerically simulated by CST Microwave Studio (Dassault Systèmes, France). According to the blue curve in Fig. 3e, the modulator has optimal excitation efficiency of about −20 dB when the resonance frequency *ω*_02_ of the parametric resonator is tuned to 306 MHz. This level of power efficiency is 29-dB better than the power efficiency of a parametric resonator without passive coupler (orange curve in Fig. 3e). Of course, for actual circuit construction, it may not be easy for the parametric resonator to precisely resonate at 306 MHz due to component value variations. But as long as the *ω*_02_ value resides between 304 MHz and 310 MHz, the excitation efficiency will only decrease by less than 0.3 dB compared to its optimal value.

### B. MODULATOR CONSTRUCTION

**FIGURE 4.**
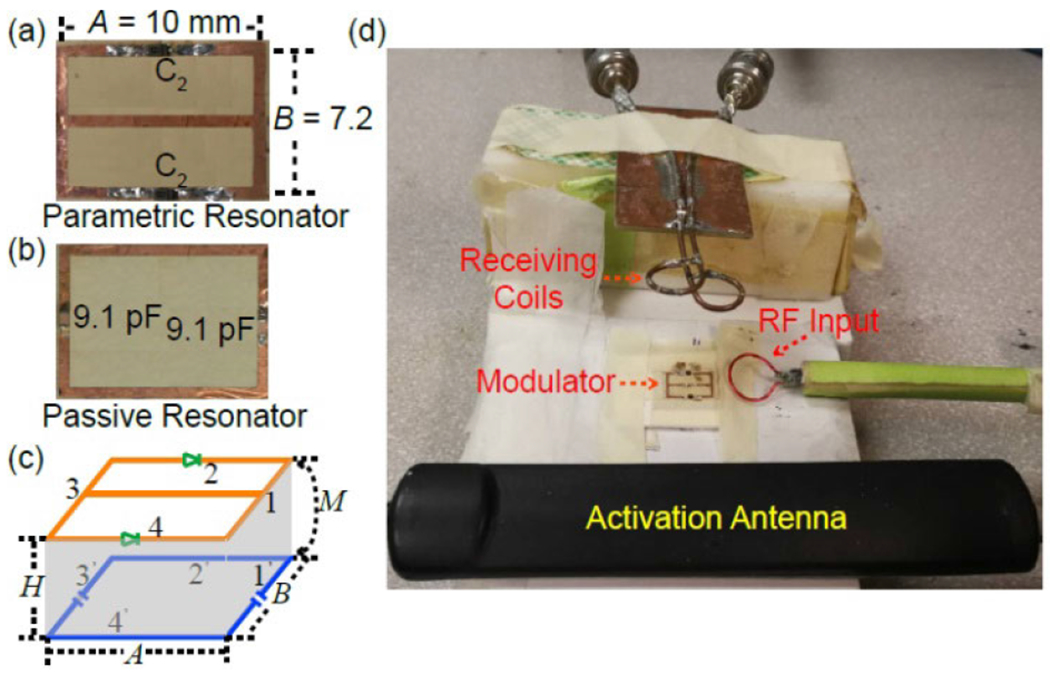
(a) The picture of a double frequency parametric resonator made of a rectangular conductor loop with a horizontal conductor in the center. The gaps in the top and bottom conductors are filled by variable capacitors *C*_2_ (MA2737600L, Panasonic, Japan). (b) The picture of a single frequency passive resonator of the same dimension but that is connected to two 9.1-pF chip capacitors connected in series. (c) The schematic representation of overlapped resonators through a polyimide substrate with thickness *H*. (d) When the activation antenna provides a pumping signal near its highest resonance frequency, the induced pumping current inside the modulator is converted into sustained oscillation currents in the two lower-frequency resonance modes. An input loop placed near the modulator can inject RF signals that will frequency modulate the oscillation signal. Upon reception by the weakly-coupled antennas, the oscillation signal is mixed down to below 1 MHz and observed on an oscilloscope via a low pass filter.

As shown in Fig. 4a, the parametric resonator is etched on a copper-clad polyimide as a rectangular conductor loop with a continuous conductor in its center. When *A* = 10 mm, *B* = 7.2 mm and *W* = 0.6 mm, the rectangular loop’s total inductance is 23.2 nH. As a result, by filling the conductor gaps on the bottom and top legs with two variable capacitors (MA2737600L, Panasonic, Japan) whose zero bias capacitance is ~22.6 pF, the parametric resonator is expected to have dipole mode resonance at 310.8 MHz. This calculated resonance frequency is in close agreement with the measured value of *ω*_02_ = 310.5 MHz on the *S*_21_ curve obtained by a pair of partially overlapped receiving coils connected to the input and output ports of a network analyzer.

To bring down the dipole mode frequency to the MR frequency at ~300 MHz, the required resonance frequency for the passive resonator can be estimated as *ω*_01_ ~ 489 MHz from Eq. (24). For this purpose, another loop inductor with the same dimension as 10 × 7.2 mm^2^ can be serially connected to two 9.1-pF capacitors. As a result, the passive resonator is estimated to have a resonance frequency at 489.9 MHz in its stand-alone configuration, which is in close agreement with the measured value at 489.6 MHz.

By adjusting the substrate thickness *H* to ~1.5 mm, the mutual inductance is estimated to be 7.9 nH. As a result, the coupled resonator pair is expected to have a lower coupled frequency at 300.2 MHz and a higher coupled frequency at 538.5 MHz, both of which are in good agreement with the measured values. Because the higher coupled frequency at 538.5 MHz is approximately the sum of the lower coupled frequency at 300.2 MHz and the butterfly resonance at 238.3 MHz, the coupled resonator can be efficiently activated by a pumping signal at 538.5 MHz to generate sustained oscillation currents at 300.2 MHz and 238.3 MHz. When the activation antenna is placed 4 cm away from the modulator, which is 4-fold the modulator’s largest dimension, the antenna only requires ~9 dBm of power to activate the modulator. This level of pumping power would produce much smaller heat compared to the 10 W/kg limit for local SAR recommended by IEC 60601-2-33.

### C. ON-BENCH CHARACTERIZATION

To validate the circuit’s capability for simultaneous down-conversion and frequency encoding, an additional circular loop is used to inject input signals at 300.21 MHz that is 10 kHz above the oscillation signal. After the modulated oscillation signal reaches the receiving coil, it is down-converted by a 300.0-MHz reference signal through a mixer (ZFY-2, Minicircuits, Brooklyn, NY) before being sampled at 3.25 MHz by a digital oscilloscope via a low-pass filter (BLP-1.9+, Minicircuits, Brooklyn, NY). Using Matlab (Mathworks, Natick, MA) for post processing, the digitally sampled data is Fourier transformed to obtain the frequency domain spectrum of the carrier wave and sidebands. Alternatively, to retrieve the input signal in the time domain, the digitally sampled data is combined with its *Hilbert* transformation to create a complex dataset. After low pass filtering by the *Hamming* window, the phase angle of this complex dataset is retrieved by the *angle* and *unwrap* functions, before the baseline is linearly corrected using the *detrend* function. Finally, the instantaneous frequency at each time point is obtained by derivatizing the phase angle using the differential filter function (*differentiatorfir*).

### D. MR IMAGING

**FIGURE 5.**
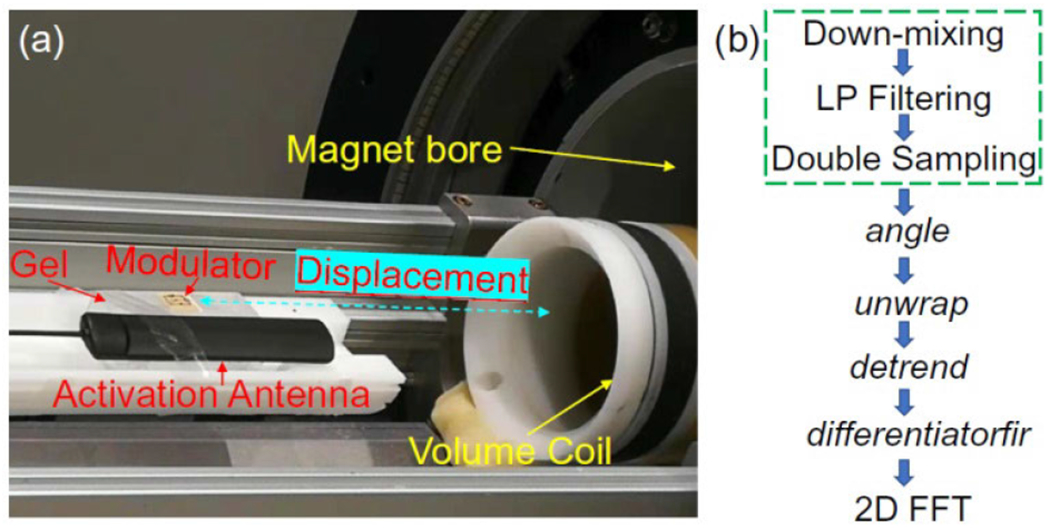
(a) In MR experiments, the modulator is placed on top of a gel phantom containing 1% agarose. The modulator, along with an activation antenna placed nearby, is inserted into a cylindrical volume coil and the entire assembly is inserted into the center of a 7T magnet. To emulate weak coupling condition, the volume coil is displaced away from the magnet’s center. For each displacement, the MR excitation power is adjusted to maintain the same effective excitation angle. During MR signal acquisition, pumping power is applied on the antenna to activate the modulator whose oscillation signal is received by the cylindrical volume coil. (b) The multi-step procedure to retrieve input RF signals from the instantaneous frequencies of the oscillation signal. For MR image reconstruction, the procedures enclosed in the dashed box are already performed by the spectrometer, so the output data can be directly processed by the five remaining steps.

To demonstrate the modulator’s capability for wireless transmission of MRI signals, the modulator is placed on top of an aqueous gel phantom containing 1%-agarose. With the activation antenna placed 4-cm away, the modulator on top of the phantom is inserted into a cylindrical volume coil (Bruker Biospin, Billerica, MA) with 70-mm inner diameter. The entire assembly is located inside a 7T horizontal bore magnet equipped with Bruker Avance III spectrometer console. Inside the scanner, the pumping signal is provided by an external RF generator (E4423, Agilent, CA) through a dipole antenna. The pumping frequency is empirically adjusted to make the oscillation frequency separated from the proton MR frequency at 7T by the image bandwidth (50 kHz in this case). To emulate weak coupling condition, the modulator is fixed at the center of a homogeneous magnet, while the volume coil is displaced from the magnet’s center. Nuclei spins are excited by the RF field produced by the volume coil and coupled through the modulator. During signal reception, the oscillation signal that is frequency modulated by MRI signals are received by the external volume coil, in a similar way as how MR signals were traditionally acquired. To retrieve MR signals from phase angles of the oscillation signal, the sampling speed should be at least the Nyquist rate (twice the image bandwidth) to obtain enough phase angles for derivatization. As shown in Fig. 5b, because the spectrometer can already perform down conversion and low-pass filtering, the *angle*, *unwrap* and *detrend* functions are directly applied on the received oscillation signal to obtain phase angle information before the differential filter is used to retrieve MR signals in the time domain. Fourier Transform is finally used to reconstruct 2D images. To evaluate the ratio between signal and noise voltages, the same image is acquired again whose magnitude is subtracted from the first image to obtain the noise map. The sensitivity map is calculated by dividing the average intensity of individual pixels with the standard deviation of the noise map:

(25) $$SNR=\frac{\left(\left|S_{1}\right|+\left|S_{2}\right|\right)/2}{std\left(\left|S_{1}\right|-\left|S_{2}\right|\right)}$$

For comparison, the ***relative SNR*** is obtained by dividing the *SNR* map obtained by the parametric oscillator with the *SNR* map obtained by a surface coil of the same dimension but with direct wired connection to the MRI console.

## IV. RESULTS

**FIGURE 6.**
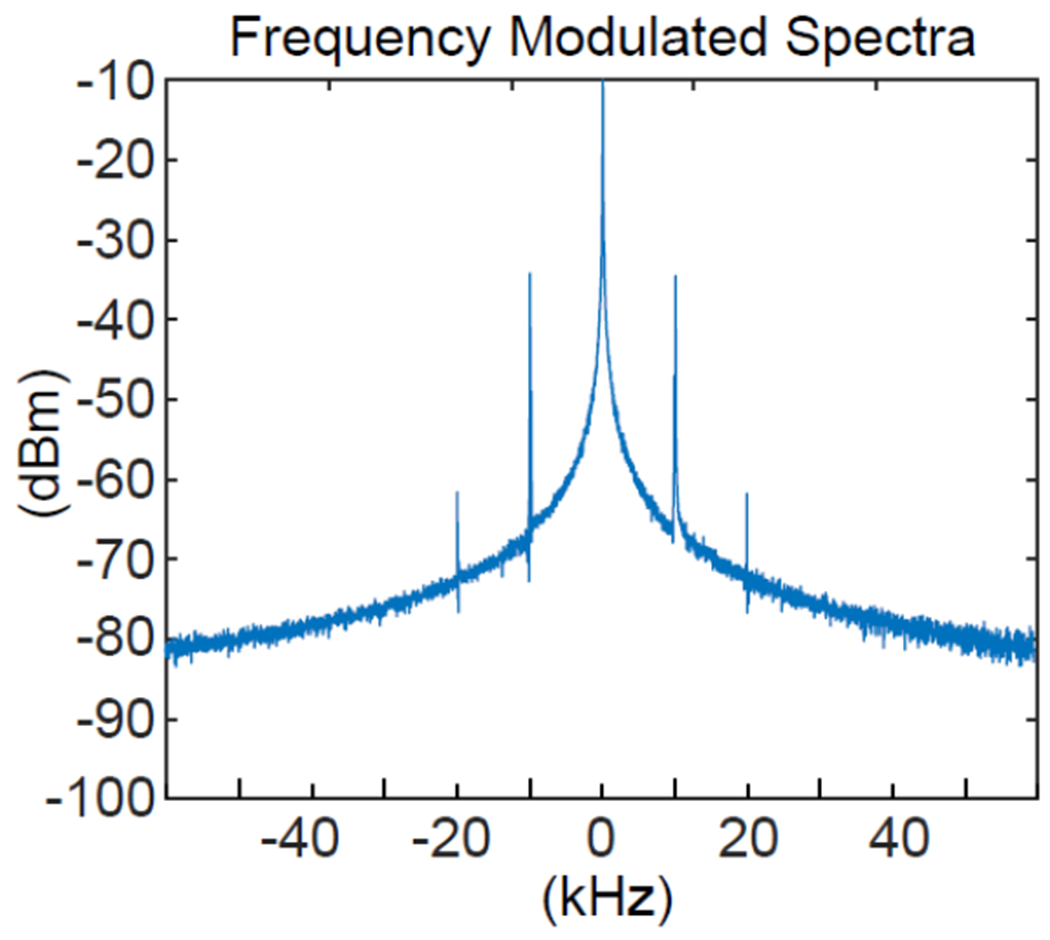
The Fourier spectrum of the oscillation signal (obtained using the setup shown in Fig. 4). An input RF signal created multiple sidebands that were separated from each other by the offset frequency between the input signal and the oscillation signal.

Using the experimental setup shown in Fig. 4, we first characterized the modulator on-bench. Fig. 6 was the Fourier spectrum of the oscillation signal obtained by the receiving coil that was separated from the modulator by a distance that was 4-fold the modulator’s largest dimension. As shown in the figure, the center oscillation peak was surrounded by multiple sidebands, each of which was separated from its neighbor by 10 kHz (the frequency offset between the input RF signal and the oscillation signal). As predicted by Eq. (11), the sideband pattern demonstrated the modulator’s capability to encode the input signal at the offset frequency, without the need for dedicated down-converters. Moreover, because the height of the first sideband was 24.2-dB lower than the center band, the frequency deviation ratio was *β* = 0.123, according to *J*_1_(*β*)/*J*_0_(*β*) ≈ *β*/2 in Eq. (12). This small *β* corresponded to narrow band modulation, thus justifying the time-domain sampling rate at twice the message bandwidth for oscillation signal demodulation, as will be mentioned below.

**FIGURE 7.**
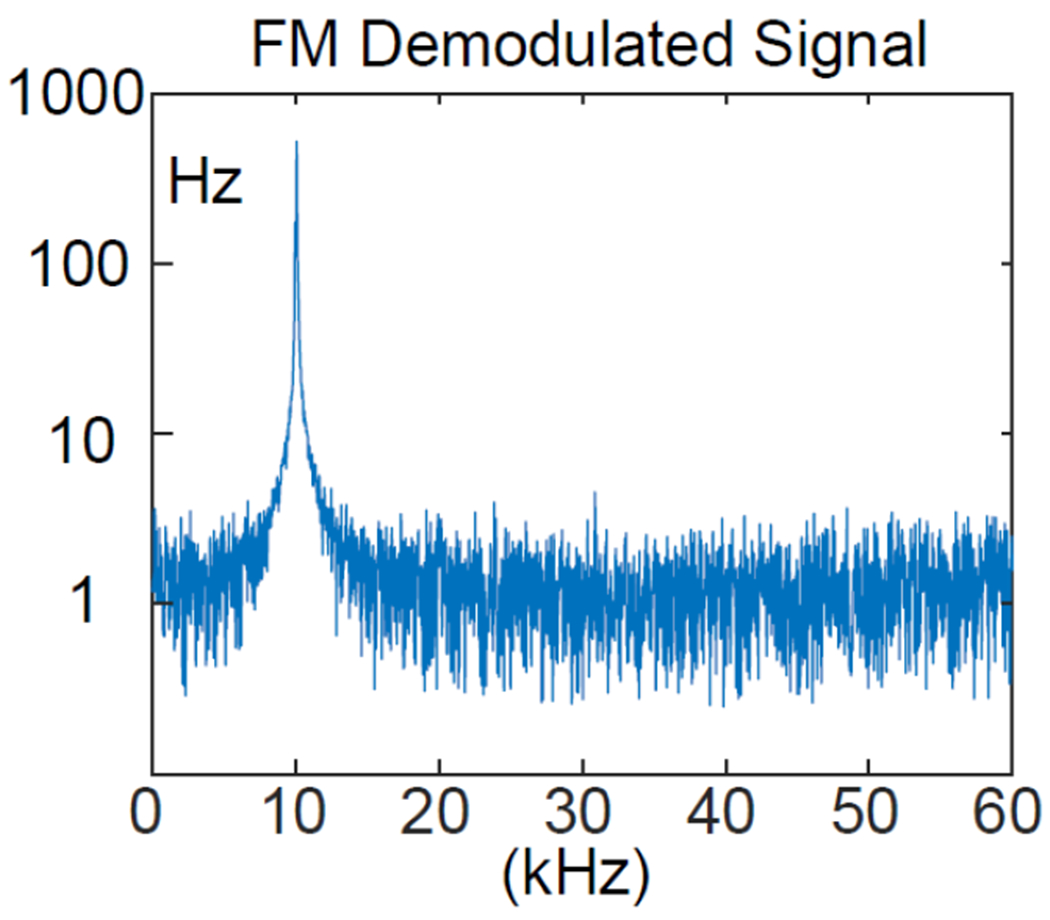
The input spectrum retrieved from the oscillation signal using the multi-step procedures described in Fig. 5b. The vertical axis represents the magnitude of frequency deviation defined in Eq. (10), while the horizontal axis represents frequency offset between the input signal and the oscillation signal.

To retrieve the input signal from the oscillation signal, we performed frequency demodulation using the procedures described in Fig. 5b. As shown in the frequency demodulated spectrum (Fig. 7), there was a sharp peak centered around 10 kHz whose height was approximately 320-fold larger than the noise floor. Therefore, the input signal could be decreased by another factor of 320 while still being observable. The almost flat noise floor also indicated that internal noise from the modulator dominates over external noise from the receiving antenna, even though the distance separation was 4-fold the modulator’s largest dimension.

**FIGURE 8.**
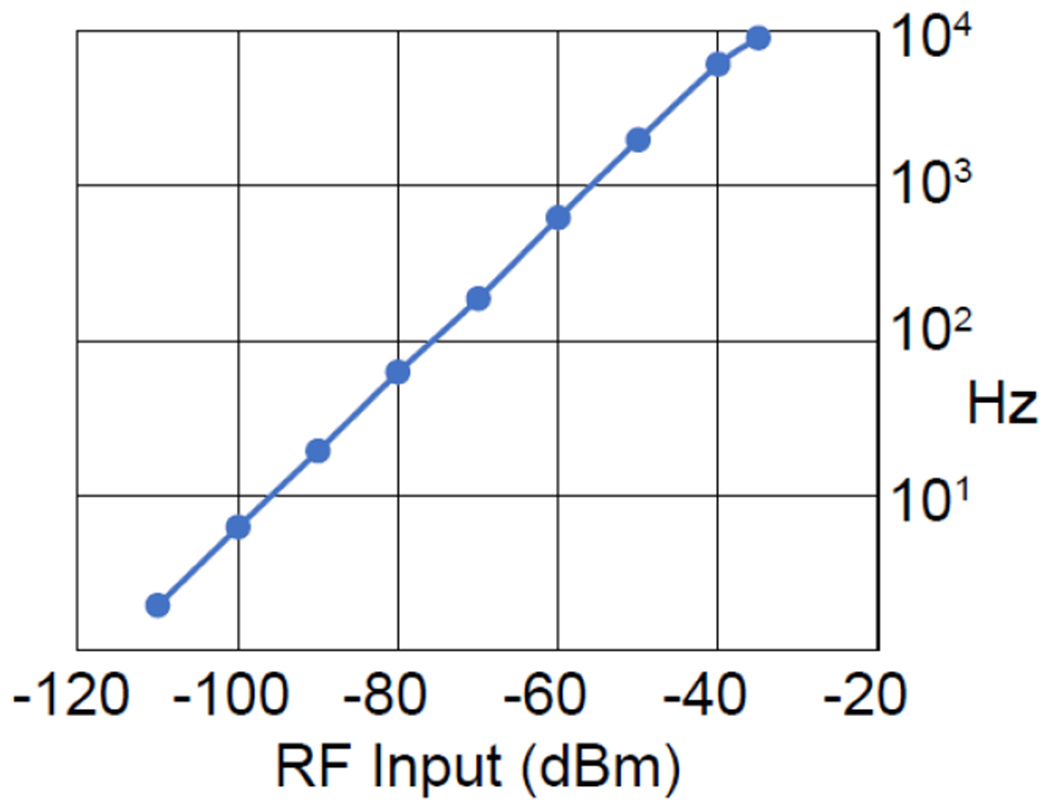
The linear relation between the input RF signal and the frequency deviation magnitude obtained by FM demodulation.

Up till now, all measurements were performed when the input signal had a power level of −60 dBm. To estimate the dynamic range of the modulator, we swept the input power and measured the peak height in the demodulated spectrum. As shown in Fig. 8, the wireless modulator had a dynamic range of 75 dB. The minimum observable input signal was −110 dBm, which was 50 dBm lower than the −60-dBm input power used previously. Fig. 8 also demonstrated the good linear relation between the input signal and the frequency deviation magnitude, thus validating the prediction of Eq. (10).

**FIGURE 9.**
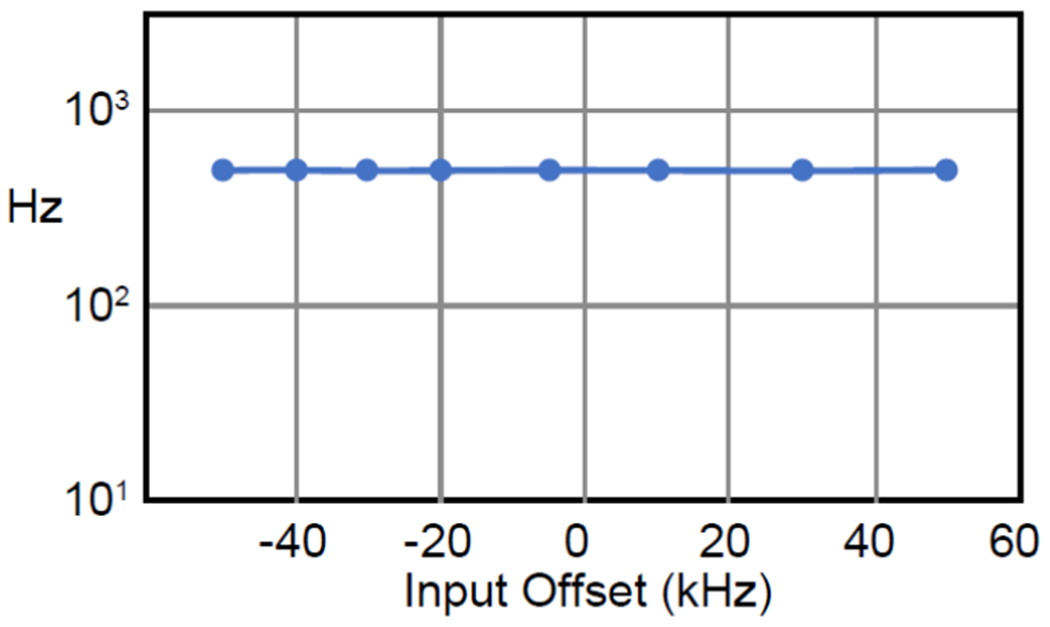
When the input signal was swept over a range of offsets from the oscillation signal, the demodulated output maintained the same magnitude of frequency deviation.

To evaluate the wireless modulator’s frequency response, we swept the input frequency and performed frequency demodulation using the procedures described in Fig. 5b. As shown in Fig. 9, the demodulated peak had the same magnitude over at least 100 kHz. This uniform frequency response of the demodulated peak was consistent with the offset-independent relation described in Eq. (10). Therefore, white noise detected by the modulator (as input signal) would lead to flat noise baseline upon FM demodulation, as will be demonstrated below.

**FIGURE 10.**
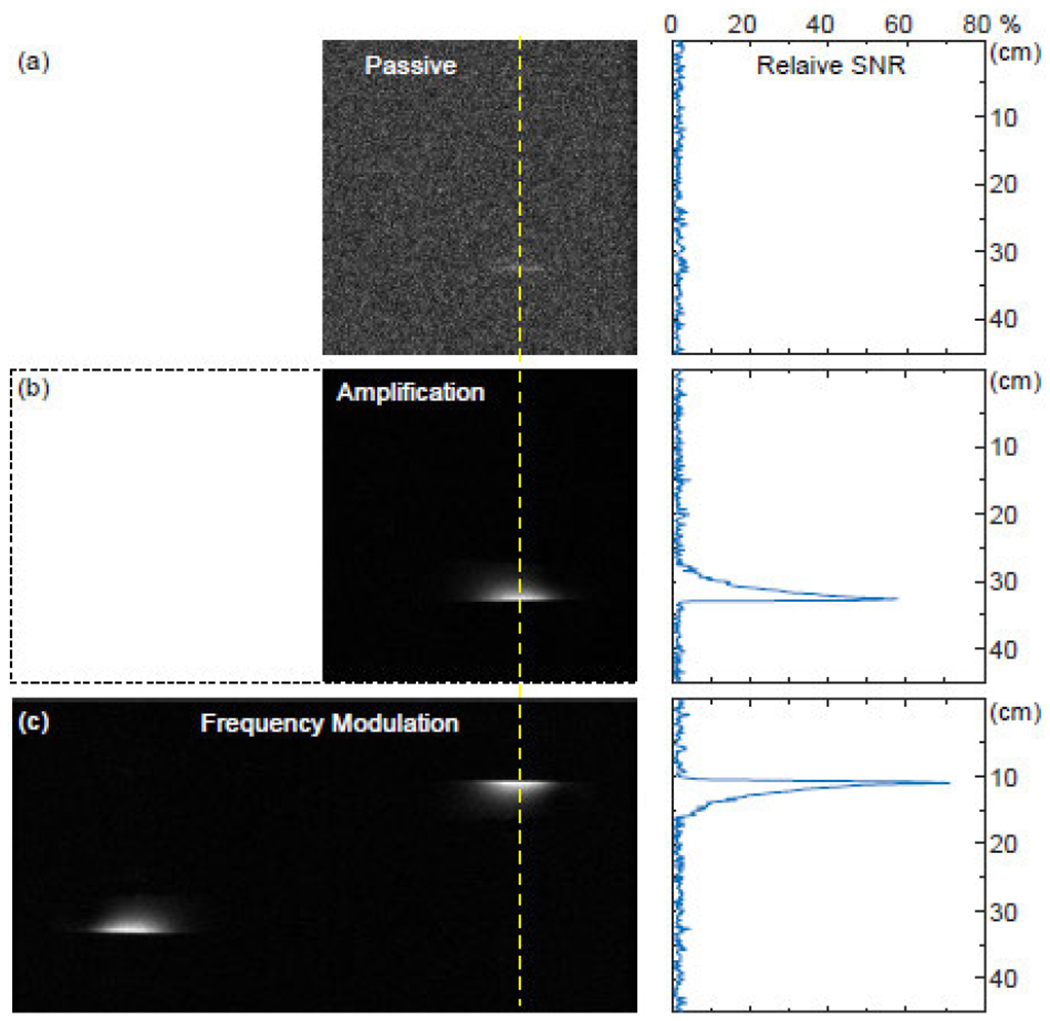
(a) When the modulator was separated from the center of volume coil by 11 cm to lay outside the volume coil’s cylindrical edge, signal acquisition by passive inductive coupling produced small observable signal. (b) When the modulator was activated by pumping signal at a power level ~0.1 dBm below its oscillation threshold, the locally detected MR signals were significantly amplified, producing a clear image with sharp intensity profile. (The intensity profile in the right most column was plotted against the yellow dashed line in MR images.) (c) When the modulator was activated by a pumping signal above its oscillation threshold, locally detected MR signals were frequency encoded onto the oscillation signal that could more effectively overcome transmission attenuation, leading to MR images with even higher *SNR*. All *SNR* values shown in the right most column were referenced against the *SNR* of a directly connected surface coil.

To demonstrate the modulator’s capability for wireless transmission of MR signals, the modulator was placed on top of a 1% agarose phantom and inserted into the center of a 7T MRI scanner. To emulate weak coupling condition, the volume coil was displaced by 11 cm away from the center of magnet. For this distance separation, the modulator already laid outside the volume coil’s cylindrical edge. To estimate the magnitude of transmission attenuation, an MR image (shown in Fig. 10a) was first reconstructed from signals that were passively relayed to the volume coil, using FOV = 45 × 45 mm^2^, acquisition matrix = 256 × 256, bandwidth = 50 kHz, effective flip angle = 5 deg, TE = 10 ms, TR = 20 ms. Compared to a conventional surface coil of the same dimension but with wired connection to the scanner console, the passively coupled modulator had a relative *SNR* of only ~2%, corresponding to 50-fold (or 34-dB) transmission attenuation under 11-cm distance separation. Subsequently, we turned on pumping signal and set its power level at ~0.1 dBm below the oscillation threshold to enable stable amplification with high gain (33 dB). As shown in Fig. 10b, there was significant increase in signal intensity because the circuit could regeneratively amplify MR input signals by parametric frequency mixing processes [26]–[29]. Finally, we increased the pumping power above the oscillation threshold and recorded the oscillation signal at doubled sampling rate. To maintain the same image resolution, the horizontal field-of-view (FOV) was correspondingly doubled to confine the original image region to the right half portion of the enlarged FOV. (As shown in Fig. 10c, when the FOV was increased to 90 × 45 mm^2^, the acquisition matrix was increased to 512 × 256 and the sampling rate was increased to 100 kHz.) By overlapping the oscillation frequency to the center of the enlarged FOV, time-domain MR signals could be retrieved by derivatizing the phase angle. The retrieved signals were subsequently Fourier Transformed to obtain the 2D image. By comparing Figs. 9c and 9b, the image obtained by frequency modulation was 24% more sensitive than the image obtained by regenerative amplification, demonstrating the better noise immunity of FM encoding. It is worth mentioning that the reconstructed image contained mirrors of the original object in both dimensions, due to the cosine-encoding relation of the message signal as described in Eq. (9).

To estimate the modulator’s effective operation range, we positioned the modulator in the center of magnet to detect the gel phantom in the same location with the same static magnetic field, while displacing the volume coil away from the magnet’s center. This would enable us to systematically vary the transmission attenuation between the modulator and the volume coil without modifying their circuit dimensions. (For each displacement, the transmission attenuation was estimated as the reciprocal of relative *SNR* of passive inductive coupling with respect to a wire-connected surface coil. Subsequently, the excitation power on the volume coil was proportionally scaled by the transmission attenuation to maintain the same effective flip angle.) As shown in Fig. 10, both the amplifier and the modulator had similar *SNR* when the attenuation was smaller than 21 dB. But the modulator could maintain constant sensitivity for attenuation up to 34 dB when the modulator already laid outside the edge of the volume coil. Even though the modulator’s sensitivity would eventually drop under very large distance separations, its *SNR* always remained higher than the amplifier, demonstrating the advantage of FM encoding of MRI signals to overcome larger transmission attenuation.

The effectiveness of the modulator can also be evaluated by its flatter image baseline along the horizontal direction. As shown in Fig. 12a, for 7.5-cm displacement that was 7.5-fold the modulator’s largest dimension, the corresponding transmission attenuation of 25-dB could be effectively compensated by the 33-dB gain provided by regenerative amplification, leading to uneven baseline that was characteristic of a high-gain regenerative amplifier with a narrowed bandwidth. Meanwhile, the image reconstructed from the modulator had a flatter baseline (Fig. 12b) when the internal noise from the modulator dominated over external noise from the volume coil, making frequency modulated detection the preferred choice. For the 15-cm displacement that was 15-fold the modulator’s largest dimension, the transmission attenuation was much larger than what regenerative amplification could overcome, leading to a flatter image baseline (Fig. 12c) that was primarily determined by the external noise. Meanwhile, the image reconstructed from the modulator had uneven baseline, due to the frequency dependent transfer function of the external noise as described in Eq. (14). Therefore, frequency modulation would provide flatter image baseline than regenerative amplification within a tolerable range of transmission attenuation. Outside this range, observation of a tilted baseline in the reconstructed image could be practically used as an indicator of too large a transmission attenuation for the frequency modulator to completely overcome.

## V. DISCUSSION

In this work, we have fabricated a triple frequency parametric oscillator that can utilize wireless pumping power provided by an activation antenna to directly encode locally detected RF signals onto its oscillation signal as the modulated carrier wave. The modulator consists of a double frequency parametric resonator with dipole and butterfly modes to sustain circuit oscillation, as well as a coupled single frequency resonator to concentrate the local pumping field. Unlike voltage-controlled oscillators that can only encode signals much slower than circuit oscillation, the parametric oscillator can combine down conversion and frequency encoding into a single stage, thus directly encoding RF inputs as frequency modulated oscillation signals for detection over larger distance separations. As predicted by Eqns. (11) and (12), the modulated oscillation signal observed by the remotely coupled receiver coil can be visualized in the frequency domain as summation of equally spaced sidebands distributed around the center carrier (Fig. 6); and the relative height of the first sideband with respect to the center peak scales proportionally to the RF input voltage *ξ_d_*. Alternatively, the input signal can be retrieved by derivatizing the modulated phase angle of the oscillation signal in the time domain (Fig. 7), leading to a constant magnitude response of decoded signals over a range of input signal offsets (Fig. 9).

Although parametric oscillators have been previously utilized for quasi-static signal sensing [5], [24], [30], this work has for the first time demonstrated the circuit’s built-in capability for simultaneous down-conversion and frequency-encoding of RF signals. Compared to on-board digitization, FM-encoded oscillation is easier to implement using a compact modulator. Because of its design simplicity, this wirelessly powered modulator is amenable to miniaturization, making itself useful for chronic operation inside confined body cavities. The modulator is a low-power device: it can be wirelessly activated by about 9-dBm of power on the activation antenna separated by a distance that is 4-fold the modulator’s largest dimension. This level of pumping power is 20 dB lower than that required for a parametric resonator in its stand-along configuration, owing to the on-resonance concentration of magnetic flux at the pumping frequency. Further reduction in the required level of pumping power would benefit from the use of loop antennas and impedance matching network that can generate larger magnetic flux per unit current [31], or benefit from the exploration of midfield power transfer schemes [32].

**FIGURE 11.**
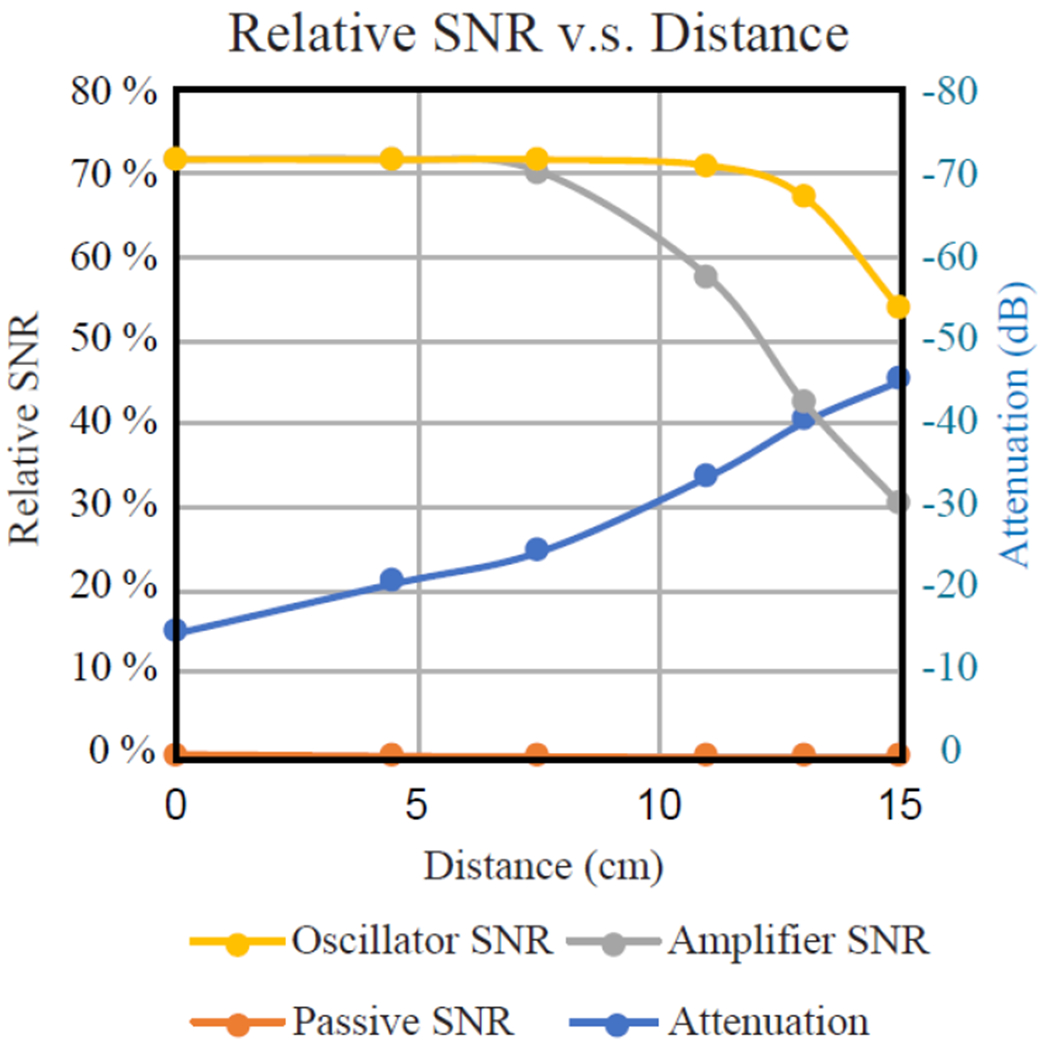
When the relative SNR of MR images was measured at different distance separations, the amplified resonator (gray) could retain constant sensitivity for transmission attenuation up to 21 dB (at 4.5 cm separation). In comparison, the frequency modulator (yellow) could retain constant sensitivity for transmission attenuation up to 34 dB (at 11 cm separation).

**FIGURE 12.**
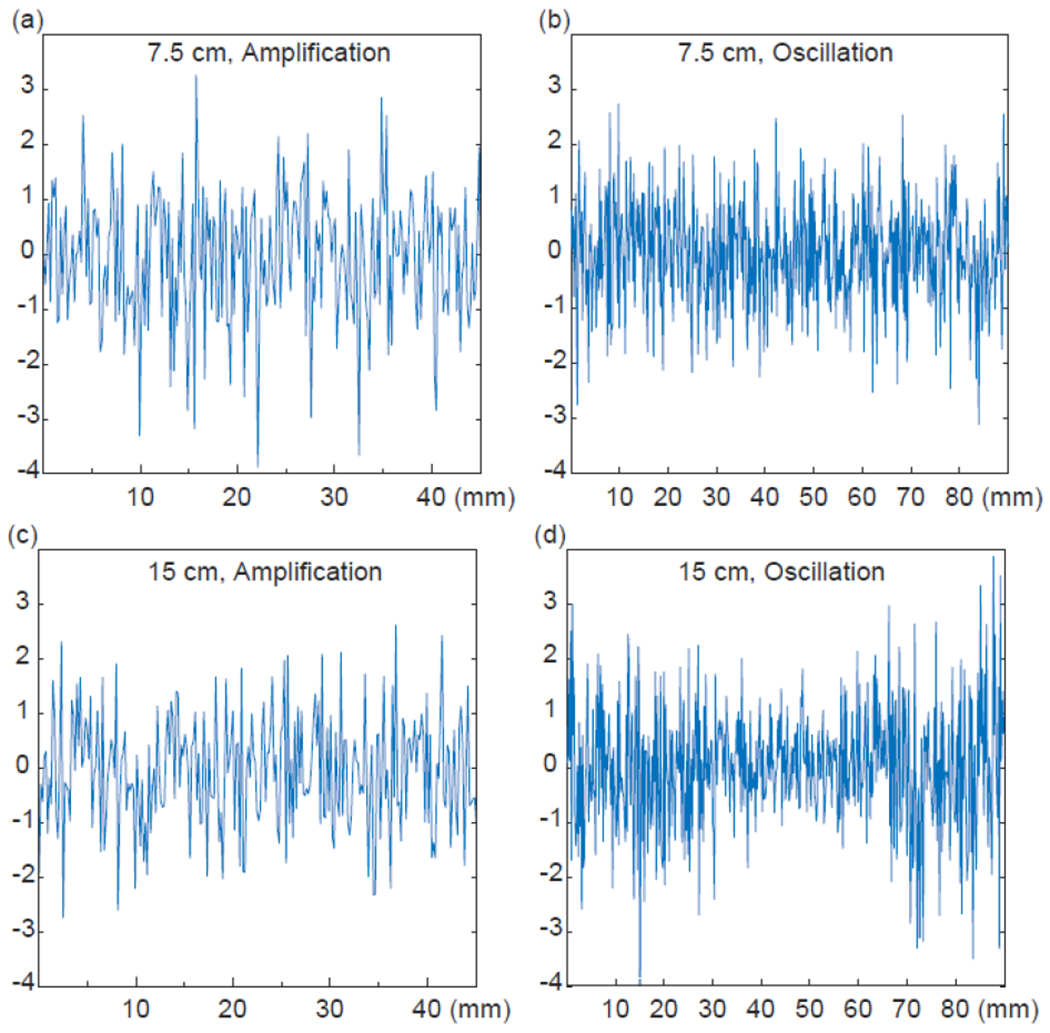
(a, c) Horizontal baselines of images acquired in the presence of regenerative amplification. (b, d) Horizontal baselines of images reconstructed from frequency modulated oscillation signal. For the 7.5-cm distance separation, the required amplifier gain to overcome transmission attenuation would make the image baseline uneven (in Fig. a) due to the amplifier’s narrowed bandwidth. Meanwhile, the baseline of the FM-demodulated image was flatter (in Fig. b) due to the modulator’s frequency invariant response to internal noise. For the 15-cm distance separation, the baseline of the amplified image was almost flat (in Fig. c), due to the predominance of external noise originating from the volume coil. Meanwhile, the baseline of the FM-demodulated image was uneven (in Fig. d), indicating too large a transmission attenuation for the frequency modulator to overcome.

To demonstrate effective MRI signal transmission under weak coupling conditions, we have systematically varied the modulator’s distance separation from the external receiver coil without modifying their circuit dimensions. This can be achieved by keeping the modulator in the magnet’s center while gradually displacing the volume coil away from the center, thus emulating increasingly large transmission attenuation. For each displacement, the overall sensitivity is affected by both the “internal” noise and the “external” noise. The internal noise comes from the dissipation current in the gel phantom and the modulator itself, while the external noise originates from the volume coil. When the modulator’s oscillation current induces a voltage *A* that is much larger than the noise voltage on the external volume coil, the internal noise will dominate and modulate the oscillation frequency in an offset independent manner as predicted by Eq. (13). This flat frequency response will lead to flat baselines in the reconstructed image (Fig. 12b). However, when the oscillation-induced voltage *A* is no longer dominant over the external noise voltage from the receiver coil, the baseline of the reconstructed images will become uneven (Fig. 12d), because Fourier transform of the time-domain derivative is equivalent to frequency domain multiplication of input signal offset as described by Eq. (14). Therefore, observation of flat baseline could be practically used to evaluate the tolerable range of transmission attenuation within which constant sensitivity can be maintained. According to the distant-dependent sensitivity plot in Fig. 11, the wireless modulator can maintain constant sensitivity even when the transmission attenuation is up to 34 dB, which is 13 dB larger than what is tolerable for regenerative amplification. To flatten the image baseline for larger transmission attenuations, it is possible to incorporate pre-emphasis filtering [33] into the parametric oscillator, or to use a chain of parametric oscillators that can frequency encode relayed signals multiple times during the wireless transmission pathway [34]. More work is going on along this direction.

In Summary, we have demonstrated the simultaneous down-conversion and frequency-encoding capability of an antenna powered parametric oscillator. This oscillator can mix the input RF signal with its own oscillation signal and modulate the oscillation signal at the offset frequency. Without the need for DC power source or multi-stage signal processors, this compact circuit can simultaneously detect, encode and broadcast locally acquired MR signals over larger distance separations, using tiny amount of wireless power. This wireless modulator can be easily inserted into body cavities or placed on skin surfaces to sensitively detect focal regions of interest in close proximity. Its broadcasting capability will also enable the modulator to wirelessly communicate with any conventional detectors that are readily available on ordinary MRI scanners, thus greatly improving MRI’s applicability for any parts of body without the need for dedicated focal coils using specialized signal interfaces. Although frequency encoding of MR signals is somewhat noisier than a directly connected surface coil, the greatly improved flexibility of this wireless modulator will justify its use inside confined space or body cavities. Owing to the better noise immunity of FM encoding, this modulator can potentially be adapted to travelling wave detection [35] where the cylindrical magnet bore can be advantageously used as a waveguide for longer range signal transmission.

## Figures and Tables

**TABLE 1.** Summary of different types of RF transducers.

Reference	[16–18]	[6–8]	[9–14]	This work
Type	Inductive coupler	Digital transmitter	Optical modulator	Parametric oscillator
Signal Processing Method	On-resonance coupling	Down-Conversion & Digitize & Encode	Direct AM	Direct FM
Carrier/input freq ratio	~1	>938	>2x10^6^	~1
Power consumption	Passive	>77 mW DC	>10 mW fiber-optic	<10 mW external RF
Dynamic Range	---	86 dB	81 dB	75 dB
Surmountable Attenuation	<10 dB	31 dB	33 dB	34 dB
